# Supplementary material for: Optimization of the Micellar-Based In Situ Gelling Systems Posaconazole with Quality by Design (QbD) Approach and Characterization by In Vitro Studies
Source: Pharmaceutics. 2022 Feb 27;14(3):526. doi: 10.3390/pharmaceutics14030526 (PMC8954786; doi:10.3390/pharmaceutics14030526)
Supplement: Supplementary file 1 [file pharmaceutics-14-00526-s001.zip › pharmaceutics-1574669-supplementary.pdf]

The equations of the studied data are listed below;

Gelling capacity= -0,82+0,033 Poloxamer 407+0,208 Poloxamer188 + 0,00618 Poloxamer407 * Poloxamer 407-0,00586 Poloxamer 188* Poloxamer188-0,00742 Poloxamer 407*Poloxamer188

Tsol/gel= 47,77+ 1,004 Poloxamer 407- 0,379 Poloxamer 188 – 0,0938 Poloxamer 407* Poloxamer 407 + 0,0426 Poloxamer 188* Poloxamer 188+ 0,0063 Poloxamer 407* Poloxamer 188

Drug Content= 113,84-0,570Poloxamer 407-0,876 Poloxamer188 – 0,0153 Poloxamer 407*Poloxamer 407-0,0094 Poloxamer 188* Poloxamer 188+ 0,0426 Poloxamer 407*Poloxamer 188

Log consistency index= 0,12-0,395 Poloxamer 407-0,240 Poloxamer 188+ 0,01845 Poloxamer 407* Poloxamer 407 + 0,00633 Poloxamer 188* Poloxamer 188+ 0,00780 Poloxamer 407* Poloxamer 188
